# Memoirs of the Royal Medical Society at Paris. Vol. II

**Published:** 1783

**Authors:** 


IV. *Memoirs of the Royal Medical Society at Paris. Vol. II.* (*Continued from p.* 49.)

*Remarks on the ill effects of corrosive sublimate in hot climates.* *By* M. de la Peyre.——This writer has experienced much more dangerous effects from the use of this remedy in the *Isle de France* and *de Bourbon* than in Europe. He has repeatedly seen it bring on pulmonary complaints, and relates some instances in which it proved fatal.—14. *Observations on different lithontriptics.* Two cases, by different correspondents, are described in this article. The first, which is by M. Fourrestier, relates to a man, who in his 78th year, began to experience symptoms of the stone, and was relieved by the use of soap and lime water, which he took for several months, from that time till his death, which happened in his 82d year, in consequence of a cold. On dissection fourteen *calculi*, of different sizes, were found in his bladder.—The other communication is by M. Bannau, concerning the good effects of *Aqua Rabelii* (a mixture of four parts parts of oil of vitriol with twelve of ſpirit of wine) in another caſe of calculus. The patient took from three to five drops of it in each glaſs of water at his meals.—15. *On the treatment of ſcrophula. By* M. Loyauté.—Hemlock and mercurials are the principal remedies recommended by this writer.—16. *On carcinomatous tumours in the rectum. By* M. Durande.—The patient, whoſe caſe is here related, fell from his horſe in 1771, and from that time complained of pain about the region of the liver, became melancholy, and had an extraordinary appetite. Some time after, he complained of hæmorrhoids, and voided fatty concretions with his ſtools. A tumour which began to form about this time in the rectum, gradually filled up that inteſtine, and was removed by ligature; but other tumours of the ſame kind were to be felt, and at length, in 1774, the patient died. On diſſection, the lower part of the rectum was found much dilated, the large inteſtines ſwelled and inflamed, and a conſiderable quantity of fluid in the cavity of the abdomen. At the upper part of the rectum were obſerved ſeveral carcinomatous tumours of different ſizes. Some were hard and white, others ſoft and of a dark colour. Above theſe the gut was ſomewhat contracted. The liver abounded with with round, white tubercles, as large as a pea, which penetrated into its fubftance, and rofe but little above its furface. In the interior part of this vifcus were others of a larger fize. At its upper part was to be feen an enormous mafs of the fame nature, white, firm, and intermixed with membranes. This mafs weighed eleven pounds, and the whole liver fourteen. The patient had never been troubled with cough, or complained of pain in his fhoulder.—17. *On the effects of an inveterate venereal virus. By* M. Macquart.—We have here an account of a woman fixty years old, who, after having twice had the *Lues venerea*, complained of violent, rheumatic pains, and was fo weak that fhe could not quit her bed. In turning herfelf in it a little before her death, one of her thigh bones broke. On diffection the brain appeared of a livid hue, the two tables of the cranium thin, the heart foft and yellowifh, all the mufcles flaccid, the blood diffolved and difcoloured, and the bones filled with a purulent and yellowifh fluid, and fo difeafed as to give way to the flighteft preffure, though they afforded no appearance of caries externally.——18. *On the efficacy of mercury in the fmall pox. By* M. Van Voenfel, *phyfician to the corps of cadets-nobles at St. Peterfburgh.*—Seventy cadets cadets having had the ſmall pox from inoculation in a favourable manner, at a time when the diſeaſe was epidemic and fatal, M. Van Voenſel aſcribes this to their having taken ſmall doſes of calomel twice a day from the time of the inſertion to that of the eruption; but from what we know of inoculation we think it likely, that the diſeaſe would have proved equally mild if the patients had taken no mercury. Some of his obſervations, however, merit attention. He has mixed calomel with the variolous fluid, and he has expoſed it to the fumes of mercury. In both inſtances no infection took place, though the ſame patients received it afterwards when inoculated with the pure fluid. He has found alſo that a mercurial plaſter applied to the puncture prevented infection, and that the variolous fluid loſt its infectious property after being frozen.—19. *On the tænia. By* M. Brunyer.—After preſcribing Storck's remedy (a mixture of ſal polychreſt, jalap, valerian, and oxymel of ſquills) without effect, in a caſe of tænia, this writer ſucceeded with the fern root. M. Raymond, of Marſeilles, we are told, has ſometimes brought away this worm with *ol. ricini*, or with olive or almond oil, giving at the ſame time the root of the mulberry-tree in powder in doſes of ʒij.—

20. *Of*

20. *Of the manner of administering squill pills in anasarca, or general dropsy, and in some other diseases of the same kind. By* M. Renaudot, *physician at St. Domingo.*—Four scruples of fresh squills and four drachms of *sal de duobus, Pharm. Paris.* (a salt of the same nature as vitriolated tartar) are directed to be rubbed together in a mortar, and made into pills of twelve grains each, which are to be slowly dried. To adults the author prescribes four and sometimes six of these pills twice a day, without exciting vomiting, which he thinks would be impossible in a cold climate. He advises diluents at the same time.——21. *On the treatment of the lues venerea. By the late* Charles Le Roy, M. D.—The principal object of this paper is to notice the good effects of a decoction, chiefly of sarsaparilla, administered by the Sicilians in cases where mercury cannot be exhibited. The author likewise speaks of the efficacy of pills, the active ingredient of which is sublimate.—22. *On the treatment of hydrophobia. By* M. Blais, *physician at Cluny.*—Of fifteen persons bit by a mad wolf, on the 8th and 9th of December 1775, four died on the 24th or 26th of hydrophobia, and three others soon after. Of eight others, who were treated with mercurials, one was seized with symptoms of hydrophobia on on the 11th of March 1776, and died on the 13th, another died of a fever ſoon after; the other ſix remain well.

[ *To be continued.* ]

